# *In vitro* screening of angiotensin converting enzyme and hydroxymethylglutaryl-coenzyme A reductase inhibitory activities in lactic acid bacteria and yeasts isolated from fermented sorghum gruels

**DOI:** 10.5114/bta/205474

**Published:** 2025-06-30

**Authors:** Praise Temilade Ozabor, Johnson Olaleye Oladele, Ilesanmi Festus Fadahunsi

**Affiliations:** 1 Department of Microbiology, Osun State University, Osogbo, Osun State, Nigeria; 2 Food Microbiology and Biotechnology Unit, Department of Microbiology, University of Ibadan, Oyo State, Nigeria; 3 Veterinary Physiology and Pharmacology, College of Veterinary Medicine and Biomedical Sciences, Texas A&M University, College Station, Texas, United States of America; 4 Phytochemistry and Toxicology Unit, Royal Scientific Research Institute, Osun State, Nigeria

**Keywords:** Trichomonascus ciferri, Lactobacillus pentosus, cardiovascular health, fermentation, spectrophotometer

## Abstract

**Background:**

3-hydroxyl-3-methylglutaryl-coenzyme A (HMG-CoA) reductase and angiotensin converting enzyme (ACE) are implicated in the pathogenesis of hyperlipidemia and hypertension, which are oxidative-stress linked conditions of public health importance. The adverse effects associated with standard clinical drugs used to inhibit these enzymes have prompted the search for alternative sources. This study was designed to investigate the *in vitro* inhibitory activities of lactic acid bacteria (LAB) and yeasts isolated from fermented sorghum gruels.

**Materials and methods:**

LAB and yeast isolates were obtained and characterized using standard methods. The HMG-CoA reductase and ACE inhibitory activities of the microbial isolates were evaluated using established protocols.

**Results:**

Screening of LAB for HMG-CoA reductase and ACE inhibitory activities revealed that at concentrations (mg/ml) of 6, 12, 24, and 48, *Lactobacillus pentosus* WSL5 exhibited the highest %HMG-CoA reductase inhibition of 3.21, 6.42, 9.17, and 12.84, with corresponding ACE inhibitory activities of 6.38, 13.17, 18.13, and 23.47, respectively. At concentrations (mg/ml) of 1, 2, 4, and 8, the yeast isolates *Trichomonascus ciferri* RSY53 demonstrated %HMG-CoA reductase inhibition of 7.71, 11.47, 14.68, and 16.97, with corresponding ACE inhibitory activities of 11.83, 20.91, 34.73, and 48.28, respectively. Furthermore, *L. pentosus* WSL5 recorded the lowest HMG-CoA reductase half-maximal inhibitory concentration (IC_50_) of 219.72 µg/ml and ACE IC_50_ of 116.22 µg/ml, while *T. ciferri* RSY53 had even lower IC_50_ values of 29.55 µg/ml for HMG-CoA reductase and 7.03 µg/ml for ACE inhibition compared to the controls.

**Conclusion:**

*L. pentosus* WSL5 and *T. ciferri* RSY53 can be considered potential starter cultures for the fermentation of functional foods aimed at supporting cardiovascular health.

## Introduction

In many African households, cereal grains account for about 77% of total food energy intake, according to the report by Onipede et al. (2021). *Ogi* is a well-documented indigenous fermented food made from cereal grains such as maize, millet, and sorghum, and is widely consumed across West Africa, particularly in Nigeria (Ijarotimi et al. 2022). The production of *ogi* involves dehulling, sorting, washing, and wet-milling. The filtrate is then allowed to ferment spontaneously for about 48–72 h (Omemu et al. 2018).

Lactic acid bacteria (LAB) and yeasts are the predominant microorganisms involved in the fermentation of cereal grains. They play key roles in acidifying the raw materials and producing organic acids such as lactic, acetic, citric, and succinic acids (Onipede et al. 2021; Ozabor et al. 2022). These organisms have also been documented in the literature to possess healthpromoting properties that make them suitable for functional food production. Some of these properties include α-amylase and α-glucosidase inhibitory activity, and the production of gamma-aminobutyric acid (GABA), as reported by Gabaza et al. (2019), Laranjo et al. (2019), Banwo et al. (2021), and Liu et al. (2021).

Hydroxylmethylglutaryl-coenzyme A reductase (HMGCoA reductase) is a key enzyme involved in cholesterol biosynthesis. Inhibiting the activity of HMG-CoA reductase is essential for reducing cholesterol synthesis by modulating low-density lipoprotein receptors (Marahatha et al. 2021). Additionally, the angiotensin-converting enzyme (ACE) regulates blood pressure by converting angiotensin I (Ang I) into angiotensin II (Ang II), a vasoconstrictor that narrows blood vessels and increases blood pressure, eventually leading to hypertension (Wang et al. 2014; Filippou et al. 2020). This occurs through the interaction of Ang II with the type I receptor (Xia et al. 2020). Together, inhibition of HMG-CoA reductase and ACE is crucial for lowering blood cholesterol and blood pressure, respectively.

“Statins” such as simvastatin and “prils” such as captopril are chemically synthesized drugs developed in the mid-1970s for treating hyperlipidemia and hypertension, respectively (Rinto et al. 2017; Chen et al. 2021). However, several studies have reported that long-term use of these drugs can cause adverse side effects, potentially leading to organ damage (Ramkumar et al. 2016; Li et al. 2020; Huang et al. 2021). This has necessitated the exploration of alternative natural sources of HMGCoA reductase and ACE inhibitors, among which LAB and yeasts have shown promise.

Some fermented food products – such as *bekasam*, and green and black teas fermented with LAB and yeasts – have been previously reported to inhibit ACE and HMG-CoA reductase (Gamboa-Gomez et al. 2016; Rinto et al. 2017). Starter culture-fermented dairy products such as yogurt and milk have also been documented by Xia et al. (2020) to inhibit ACE activity. According to Banwo et al. (2022), the inhibitory effects observed in LAB and yeasts may be due to the production of bioactive peptides released into the fermenting matrix during fermentation. Microbial genera reported in previous studies to contribute to HMG-CoA reductase and ACE inhibition include *Lactococcus, Lactobacillus, Candida, Xanthomonas, Streptomyces, Bacillus*, and *Actinomadura* (Rinto et al. 2017; Li et al. 2020).

Although several studies in Nigeria have reported HMG-CoA reductase and ACE inhibitory activities by botanicals, to the best of our knowledge, this is the first report documenting such inhibitory activities by LAB and yeasts isolated in Nigeria as alternative natural inhibitors without known side effects.

Therefore, this study aimed to investigate the *in vitro* inhibition of HMG-CoA reductase and ACE by LAB and yeasts isolated from spontaneously fermented sorghum gruels, with potential application as starter cultures in the fermentation of functional foods or nutraceuticals.

## Materials and methods

### Collection and fermentation of sorghum grains

Spontaneously fermented sorghum *ogi* was prepared according to the method described by Ozabor et al. (2022). White and red sorghum grains were purchased from Olu-Ode Market in Osogbo, Osun State, Nigeria, and identified in the Department of Plant Biology, Osun State University, Osogbo. The grains were packaged in low-density polyethylene bags. Foreign materials such as dirt, broken grains, and stones were removed manually, and the grains were thoroughly washed with potable water. Five hundred grams (500 g) of each grain sample were steeped in distilled water for 2 days (48 h), wet-milled, sieved, and allowed to ferment spontaneously for 72 h, following the procedure described by Ozabor et al. (2022).

### Serial dilution

The fermented sorghum was serially diluted tenfold. Ten grams (10 g) of each fermentate were weighed and transferred into sterile test tubes containing 90 ml of sterile distilled water to form the stock solution. One (1) ml of the stock was then dispensed into nine test tubes, each containing 9 ml of sterile distilled water, and arranged in test tube racks. Dilution factors of 10^5^ and 10^7^ were spread-plated onto sterile Petri dishes containing MRS agar and YEA for the enumeration of LAB and yeasts, respectively (Ojokoh et al. 2014).

### Isolation of LAB and yeasts

Lactic acid bacteria and yeasts were cultured on de Man, Rogosa, and Sharpe (MRS) agar and yeast extract agar (YEA), respectively (HiMedia Laboratories, Kennett Square, USA). LAB cultures were incubated anaerobically at 37°C for 18–24 h while yeast cultures on YEA were incubated aerobically at 30°C for 18–24 h.

### Molecular identification of LAB and yeasts

The isolated LAB and yeasts were identified molecularly using polymerase chain reaction (PCR) and Sanger sequencing techniques. Genomic DNA was extracted following the manufacturer’s instructions using the Quick-DNA^TM^ Miniprep Plus Kit (catalog nos. D4068 and D4069, Zymo Research). The Lb F/R primers (5′-GAGTTTGATCCTGGCTCAG-3′/5′-AGAAAG-GAGGTGATCCAGCC-3′) were used for LAB identification, while ITS4 and ITS5 primers (5′-TC-CTCCG-CTTATTGATATGC-3′/5′GGAAGTAAAAGTCGTAACAA-GG-3′) were used for yeast identification.

The PCR protocol for LAB was: 95°C for 3 min (initial denaturation), followed by 94°C for 30 s (denaturation), 50°C for 30 s (annealing), 72°C for 90 s (extension), and a final elongation at 72°C for 5 min. The PCR protocol for yeast isolates was: 95°C for 10 min (initial denaturation), followed by 94°C for 30 s (denaturation), 55°C for 30 s (annealing), 72°C for 1 min (extension), and final elongation at 72°C for 7 min (Angelov et al. 2017; Tilahun et al. 2018; Banwo et al. 2021).

The evolutionary distances for the phylogenetic trees were computed using the Maximum Composite Likelihood method.

The nucleotide sequences of the identified LAB and yeast isolates were submitted to the National Center for Biotechnology Information (NCBI) database, and the assigned accession numbers are as follows: *Candida tropicalis* RSY43 (PP110435); *Cryptococcus* sp. RSY48 (PP110438); *Cryptococcus albidus* RSY51 (PP110440); *Naganishia albida* WSY40 (PP110441); *Lactobacillus pentosus* WSL5 (PP115584); *L. plantarum* RSL1 (PP115585); and *L. delbrueckii* RSL11 (PP115587).

#### Screening for hydroxymethylglutaryl coenzyme A (HMG-CoA) reductase inhibitory activities by LAB and yeast strains

The HMG-CoA reductase inhibitory activity of the LAB and yeast isolates was determined based on spectrophotometric measurements. Twenty-four-hour-old cultures of LAB and yeasts were aseptically transferred into Eppendorf tubes, lyophilized, and weighed. Each sample was mixed with a reaction mixture containing nicotinamide adenine dinucleotide phosphate (NADPH, 400 µM), HMG-CoA substrate (400 µM), and 100 mM potassium phosphate buffer (pH 7.4) supplemented with potassium chloride (120 mM), ethylenediaminetetraacetic acid (EDTA, 1 mM), and dithiothreitol (DTT, 5 mM). This was followed by the addition of HMG-CoA reductase (2 µl). The reaction mixture was incubated at 37°C, and absorbance was measured at 340 nm after 10 min. Simvastatin (Sigma-Aldrich Co.) was used as a positive control and distilled water as a negative control (Dewanti et al. 2017; Jaipal et al. 2022).

The percentage of HMG-CoA reductase inhibition was calculated using the following formula:


$$\text{Percentage inhibition}=\frac{\Delta \text{absorbance control}–\Delta \text{absorbance sample}}{\Delta \text{absorbance control}}\times 100\%$$

### Screening for angiotensin-converting enzyme (ACE) inhibitory activities by LAB and yeast strains

The ACE inhibition assay was conducted using a spectrophotometric method. Twenty-four-hour-old cultures of LAB and yeasts were aseptically transferred into Eppendorf tubes, lyophilized, and weighed. Stock solutions were prepared and serially diluted in 0.1 M potassium phosphate buffer containing 0.2 M sodium chloride (NaCl) to obtain different concentrations.

Fifty microliters (50 µl) of each sample and 50 µl of ACE (0.05 mU/µl) were mixed and preincubated at 37 °C for 10 min. Then, 150 µl of 6.5 mM hippuryl-histidiyl-leucine (HHL) solution was added, and the reaction mixture was incubated at 37°C for 60 min. The reaction was terminated by adding 250 µl of 1 M HCl. The reaction product, hippuric acid (HA), was extracted by adding 1.5 ml of ethyl acetate. The mixture was centrifuged at 3000 rpm for 15 min, and 1 ml of the supernatant was transferred to a 5 ml test tube for ethyl acetate removal. The tubes were placed in a water bath at 80°C until complete evaporation. The resulting residues were re-dissolved in 1 ml of deionized water before UV-VIS measurement at 228 nm. Absorbance was measured in duplicate using deionized water as the blank (Geogalaki et al. 2017; Ahmed et al. 2018).

#### Statistical analysis

Values are presented as means ± standard error from triplicate measurements at four independent concentrations. An asterisk (*) indicates statistical significance compared to the control at *p* < 0.05. Differences in means were analyzed using one-way ANOVA (analysis of variance) at α = 0.05.

### Results

#### The ACE and HMG-CoA reductase inhibitory activities of LAB

**Figure 1:**
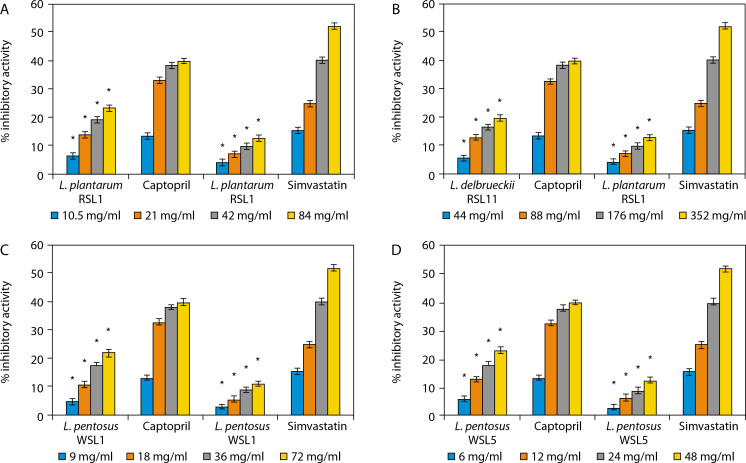
**A**) Angiotensin converting enzyme (ACE) and 3-hydroxyl-3-methylglutaryl-coenzyme A (HMG-CoA) reductase inhibitory activity produced by *L. plantarum* RSL1. *Significant when compared with control at *p* < 0.05. **B**) ACE and HMG-CoA reductase inhibitory activity produced by *L. delbrueckii* RSL11. *Significant when compared with control at *p* < 0.05. **C**) ACE and HMG-CoA reductase inhibitory activity produced by *L. pentosus* WSL1. *Significant when compared with control at *p* < 0.05. **D**) ACE and HMG-CoA reductase inhibitory activity produced by *L. pentosus* WSL5. *Significant when compared with control at *p* < 0.05

The percentage ACE and HMG-CoA reductase inhibitory activities of the LAB isolates used in this study are shown in Figure 1. The isolates include *Lactobacillus plantarum* RSL1 (Figure 1A), *L. delbrueckii* RSL11 (Figure 1B), *L. pentosus* WSL1 (Figure 1C), and *L. pentosus* WSL5 (Figure 1D).

It was observed that increasing the concentrations of LAB correspondingly increased both ACE and HMG-CoA reductase inhibitory activities. At concentrations of 6, 12, 24, and 48 mg/ml, *L. pentosus* WSL5 exhibited the highest ACE inhibitory activities of 6.38, 13.17, 18.13, and 23.47%, respectively, along with HMG-CoA reductase inhibitory activities of 2.21, 6.42, 9.17, and 12.84%, respectively.

Conversely, *L. delbrueckii* RSL11, at concentrations of 44, 88, 176, and 352 mg/ml, showed the lowest ACE inhibitory activities of 5.72, 13.17, 16.60, and 20.04%, respectively, and HMG-CoA reductase inhibitory activities of 4.59, 7.33, 10.10, and 13.30%, respectively.

#### The ACE and HMG-CoA reductase inhibitory activities of yeasts

**Figure 2:**
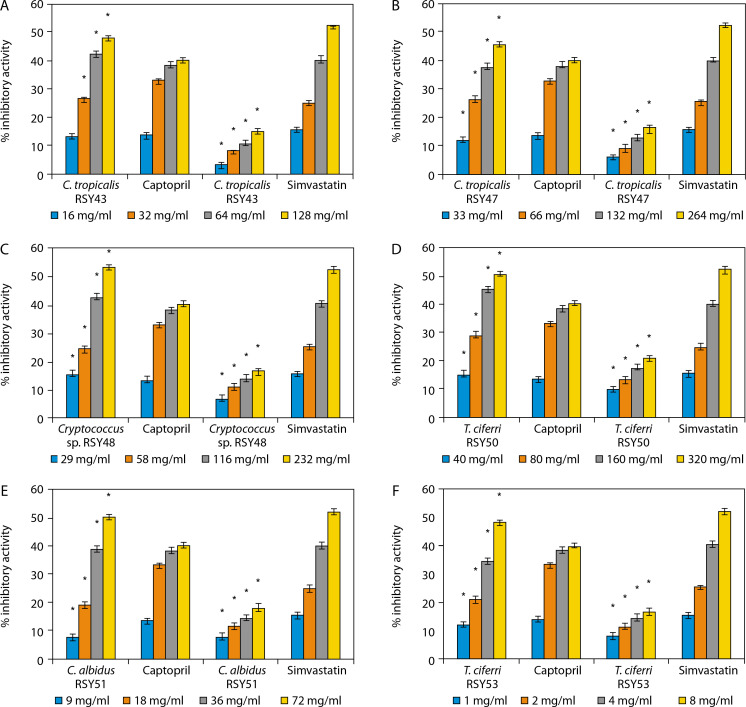
**A**) Angiotensin converting enzyme (ACE) and 3-hydroxyl-3-methylglutaryl-coenzyme A (HMG-CoA) reductase inhibitory activities of *C. tropicalis* RSY43. *Significant when compared with control at *p* < 0.05. **B**) ACE and HMG-CoA reductase inhibitory activities of *C. tropicalis* RSY47. *Significant when compared with control at *p* < 0.05. **C**) ACE and HMG-CoA reductase inhibitory activities of *Cryptococcus* sp. RSY48. *Significant when compared with control at *p* < 0.05. **D**) ACE and HMG-CoA reductase inhibitory activities of *T. ciferri* RSY50. *Significant when compared with control at *p* < 0.05. **E**) ACE and HMG-CoA reductase inhibitory activities of *C. albidus* RSY51. *Significant when compared with control at *p* < 0.05. **F**) ACE and HMG-CoA reductase inhibitory activities of *T. ciferri* RSY53. *Significant when compared with control at *p* < 0.05

The percentage ACE and HMG-CoA reductase inhibitory activities of the selected yeast strains are presented in Figure 2. The strains include *Candida tropicalis* RSY43 (Figure 2A), *C. tropicalis* RSY47 (Figure 2B), *Cryptococcus* sp. RSY48 (Figure 2C), *C. albidus* RSY51 (Figure 2D), *Trichomonascus ciferri* RSY53 (Figure 2E), and *C. tropicalis* WSY31 (Figure 2F).

An increase in yeast concentration resulted in a corresponding increase in %ACE and HMG-CoA reductase inhibitory activities. At concentrations of 1, 2, 4, and 8 mg/ml, *T. ciferri* RSY53 exhibited the highest ACE inhibitory activities of 11.83, 20.91, 34.73, and 48.28%, respectively, alongside HMG-CoA reductase inhibitory activities of 7.71, 11.47, 14.68, and 16.97%, respectively. Conversely, *C. tropicalis* WSY31 showed the lowest ACE inhibitory activities of 6.87, 16.79, 34.16, and 40.83%, and HMG-CoA reductase inhibitory activities of 6.8, 10.55, 13.30, and 16.06% at concentrations of 37, 74, 148, and 296 mg/ml, respectively.

**Figure 3:**
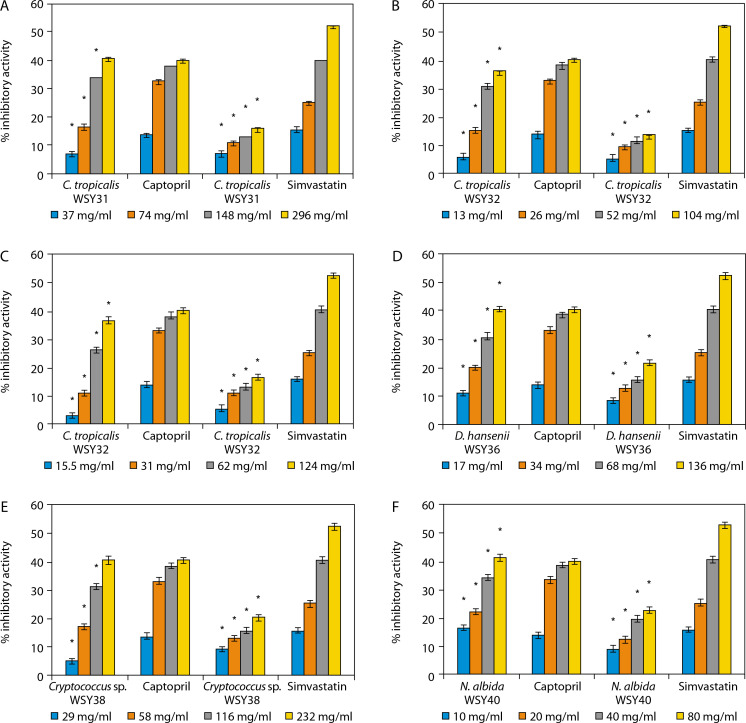
**A**) Angiotensin converting enzyme (ACE) and 3-hydroxyl-3-methylglutaryl-coenzyme A (HMG-CoA) reductase inhibitory activities of *C. tropicalis* WSY31. *Significant when compared with control at *p* < 0.05.**B)** ACE and HMG-CoA reductase inhibitory activities of *C. tropicalis* WSY32. *Significant when compared with control at *p* < 0.05.**C)** ACE and HMGCoA reductase inhibitory activities of *C. tropicalis* WSY35. *Significant when compared with control at *p* < 0.05.**D)** ACE and HMG-CoA reductase inhibitory activities of *D. hansenii* WSY36. *Significant when compared with control at *p* < 0.05.**E)** ACE and HMG-CoA reductase inhibitory activities of *Cryptococcus* sp. WSY38. *Significant when compared with control at *p* < 0.05. **F**) ACE and HMG-CoA reductase inhibitory activities of *N. albida* WSY40. *Significant when compared with control at *p* < 0.05

Notably, the yeast isolates demonstrated higher ACE inhibitory activity than the standard reference drug (captopril), and their HMG-CoA reductase inhibitory activity also increased in a concentration-dependent manner (Figure 3). The yeast strains presented in Figure 3 include *C. tropicalis* WSY31 (Figure 3A), *C. tropicalis* WSY32 (Figure 3B), *C. tropicalis* WSY35 (Figure 3C), *Debaryomyces hansenii* WSY36 (Figure 3D), *Cryptococcus* sp. WSY38 (Figure 3E), and *Naganishia albida* WSY40 (Figure 3F).

Half-maximal inhibitory concentration (IC_50_) connotes the concentration at which LAB, yeasts, or standard controls (simvastatin and captopril) inhibit HMG-CoA reductase or ACE activity by 50%. Lower IC_50_ values indicate lower systemic toxicities which implies that when the IC_50_ value (s) is low, inhibitory activity by the microbial isolates can be achieved at a very minimum concentration. Thus, establishing the effectiveness of the LAB and yeasts.

Among the LAB, *L. pentosus* WSL5 recorded the lowest HMG-CoA reductase IC_50_ value of 219.72 µg/ml and ACE IC_50_ value of 116.22 µg/ml, while *L. delbrueckii* RSL11 showed the highest IC_50_ values – 1700.47 µg/ml for HMG-CoA reductase and 1057.49 µg/ml for ACE.

Among the yeast organisms, the lowest HMG-CoA reductase inhibitory IC_50_ value of 29.55 µg/ml with ACE inhibitory IC_50_ value of 7.03 µg/ml was obtained from *T. ciferri* RSY53 while *C. tropicalis* WSY31 was recorded to have the highest HMG-CoA reductase inhibitory IC_50_ value of 1296.77 µg/ml with an ACE inhibitory IC_50_ value of 331.93 µg/ml as documented in Table 1.

**Table 1:** Half-maximal inhibitory concentration (IC_50_) values for 3-hydroxyl-3-methylglutaryl-coenzyme A (HMG-CoA) reduc-tase and angiotensin converting enzyme (ACE) inhibitions produced by the selected lactic acid bacteria (LAB) **and yeas**t strains

S/No	Organism name	HMG-CoA reductase IC_50_ (µg/ml)	ACE IC_50_ (µg/ml)
1	*Lactobacillus plantarum* RSL1	421.12	209.22
2	*Lactobacillus delbrueckii* RSL11	1700.47	1057.91
3	*Lactobacillus pentosu*s WSL1	381.21	175.49
4	*Lactobacillus pentosus* WSL5	219.72	116.22
5	*Candida tropicalis* RSY47	1073.42	272.92
6	*Cryptococcus sp.* RSY48	1002.51	198.71
7	*Trichomonascus ciferri* RSY50	1040.69	272.55
8	*Cryptococcus albidu*s RSY51	46.28	63.33
9	*Trichomonascus ciferri* RSY53	29.55	7.03
10	*Candida tropicalis* RSY43	481.76	120.67
11	*Candida tropicalis* WSY31	1296.77	331.93
12	*Candida tropicalis* WSY32	553.08	137.73
13	*Candida tropicalis* WSY35	485.76	156.87
14	*Debaryomyces hansenii* WSY36	391.20	163.68
15	*Cryptococcus sp.* WSY38	811.12	269.75
16	*Naganishia albida* WSY40	206.37	93.76
**Simvastatin** (control)		990.10	–
**Captopril** (control)		–	469.79

### Discussion

The search for natural sources of bioactive peptides with potential therapeutic applications has gained significant attention in recent years. Among the diverse microbial world, LAB and yeasts have emerged as promising candidates, exhibiting bioactivities that could contribute to the development of novel functional foods and biotherapeutic agents. ACE plays an important role in blood pressure regulation through its involvement in the renin-angiotensin-aldosterone system, while HMGCoA reductase is a key enzyme in cholesterol biosynthesis (Xia et al. 2020; Jaipal et al. 2022). Therefore, the inhibitions of HMG-CoA reductase and ACE is a key approach targeted towards the treatment of hyperlipidemia and hypertension. Although there are clinical drugs for the inhibitions of HMG-CoA reductase and ACE, studies have shown that they produce deleterious effects that can lead to organ damage (Daliri et al. 2018; Galli et al. 2019), hence, the need for natural, novel and nontoxic sources of HMG-CoA reductase and ACE inhibitors.

Therefore, this study contributes valuable insight into the HMG-CoA reductase and ACE inhibitory activities exhibited by LAB and yeasts isolated from *ogi*, a spontaneously fermented sorghum gruel commonly consumed in Nigeria. The findings support the potential application of these isolates as starter cultures in the production of natural, safe, and novel functional foods and/or nutraceuticals specifically targeted at managing cholesterol and blood pressure through enzyme inhibition.

In this study, it was observed that as the concentration of LAB increased, both ACE and HMG-CoA reductase inhibitory activities also increased. This observation aligns with the earlier findings of Li et al. (2020). ACE inhibitory activity has previously been recorded in *L. plantarum, Bifidobacterium animalis*, and *Streptococcus thermophilus* used as starter cultures for milk fermentation (Dewanti et al. 2017). Daliri et al. (2018) also reported ACE inhibitory activities in *Pediococcus acidilactici* SDL1414, *L. plantarum* JDFM44, *Enterococcus faecium* SC54, *P. acidilactici* DM9, *Lactobacillus brevis* SDL1411, *Pediococcus pentosaceus* SDL1409, and *Lacticaseibacillus rhamnosus* JDFM6 isolated from whey proteins and used as starter cultures for nutraceutical production – results consistent with the findings of this present study.

The HMG-CoA reductase inhibitory activity of LAB has also been documented by Tsai et al. (2014), who reported LAB strains with cholesterol-lowering properties, supporting the results of this work. ACE-inhibiting species of *Lactobacillus* sp., *Enterococcus* sp., *Streptococcus* sp., and *Lactococcus* sp. have also been isolated from traditional Greek dairy products by Georgalaki et al. (2017). In addition, Biswas et al. (2014), Rodriguez et al. (2019), and Xia et al. (2020) reported ACE inhibition from aqueous tomato extracts, LAB from blue corn hydrolysates, and milk products fermented with *L. plantarum* QS670, respectively.

Moreover, the study by Dewanti et al. (2017) reported that *L. acidophilus* isolated from fermented *bekasam* in Indonesia inhibited HMG-CoA reductase, which aligns with the present findings. Fan et al. (2022) further investigated ACE and HMG-CoA reductase inhibitory activities in microbial isolates, substantiating the trends observed in this study.

ACE inhibition by LAB has also been reported by Chen et al. (2021) and Glazunova et al. (2022) from plant extracts, *L. delbrueckii* LB100, and *L. lactis* AM1 used as starter cultures for dairy fermentation. HMG-CoA reductase inhibition in *Gryllus bimaculatus* fermented by *Bacillus* sp. and *Lactobacillus* strains was reported by Jang and Kim (2021). Similarly, Yadav et al. (2019) documented HMG-CoA reductase inhibition by *L. rhamnosus* MTCC:5957 and MTCC:5897 employed as starter cultures for dairy fermentation targeted at ameliorating hyperlipidemia.

The ACE and HMG-CoA reductase inhibitory activities demonstrated by the yeast strains were also observed to increase in a concentration-dependent manner. Previous studies by Lachenmeier et al. (2012), Ni et al. (2012), Burke (2015), and Jaipal et al. (2022) have reported HMG-CoA reductase inhibitory activity from *Monascus* sp., *M. purpureus*, and *Prosopis cineraria* extract. Red yeast rice, fermented by *Monascus* species, has been widely consumed as a food supplement for managing hyperlipidemia. These findings are consistent with the results obtained in the present study.

Similarly, Pavon et al. (2013) and Georgalaki et al. (2017) previously reported HMG-CoA reductase inhibitory activity from fungi, demonstrating their potential as biotherapeutic agents in animal models. Furthermore, Hipol et al. (2020) and Rahmi et al. (2022) documented HMG-CoA reductase activity in *Cryptococcus* sp., *Trichosporon* sp., *Colletotrichum* sp., and endophytic fungi isolated from lemongrass.

In addition, studies by Mirzaei et al. (2016) and Li et al. (2022) reported antioxidant and ACE inhibitory activities from *Saccharomyces cerevisiae* and the mushroom *Stropharia rugosoannulata*, respectively – supporting the current findings. Ansor et al. (2013) and Rai et al. (2015) also documented ACE-inhibitory peptides from the mycelia of *Ganoderma lucidum* and fermented dairy products, both targeted at reducing oxidative stress and preventing cardiovascular diseases such as hypertension and hyperlipidemia. Other sources of ACE and HMG-CoA reductase inhibition reported in the literature include algae (Ko et al. 2023). Although such inhibitory effects have predominantly been reported from botanicals (Chakraborty and Roy 2021; Huang et al. 2021), the use of food-grade microorganisms as novel inhibitors is an emerging area of interest.

The half-maximal inhibitory concentration (IC_50_) represents the concentration at which LAB and yeast starter cultures inhibit ACE and HMG-CoA reductase by 50%. Lower IC_50_ values indicate reduced systemic toxicity, as effective enzyme inhibition is achieved at lower concentrations. According to Daliri et al. (2018), IC_50_ values (%) of 19.78, 65.53, 70.50, 96.70, 1280.00, 2070.00, and 2130.00 were obtained from *P. acidilactici* SDL1414, *L. plantarum* JDFM44, *Enterococcus faecalis* SC54, *P. acidilactici* DM9, *L. brevis* SDL1411, *P. pentosaceus* SDL1409, and *L. rhamnosus* JDFM6, respectively, all isolated from whey proteins – results that align with this study.

Xia et al. (2020) also reported similar IC_50_ values from *L. plantarum* isolated from whey proteins in milk. Comparable results were observed in the study by Rinto et al. (2017), which documented IC_50_ values from *Lactobacillus* species isolated from *bekasam*. In support, Glazunova et al. (2022) reported similar ACE inhibitory IC_50_ values from milk fermented with co-cultures of *L. delbrueckii, Lacticaseibacillus paracasei*, and *Streptococcus thermophilus*.

Therefore, due to the lower IC_50_ values obtained from the yeasts and LAB isolates documented in this study when compared to the controls (captopril and simvastatin), especially *T. ciferri* RSY53 and *L. pentosus* WSL5, it can be inferred that these microbial strains are better and novel inhibitors of HMG-CoA reductase and ACE than the known clinical drugs (simvastatin and captopril) since high enzymes inhibition can be achieved at low concentrations of the LAB and yeast functional starter cultures.

### Conclusions

Taken together, the collective evidence from this study underscores the potential of the isolated LAB and yeast strains in inhibiting ACE and HMG-CoA reductase activities. Among the isolates, the LAB strain *Lactobacillus pentosus* WSL5 and the yeast strain *Trichomonascus ciferri* RSY53 exhibited the highest enzyme inhibitory activities, accompanied by the lowest IC_50_ values. These findings suggest that *L. pentosus* WSL5 and *T. ciferri* RSY53 could serve as promising starter cultures for fermentation processes aimed at producing functional foods designed to ameliorate hyperlipidemia and hypertension through the inhibition of HMG-CoA reductase and ACE.

Furthermore, future research can be directed towards developing functional food formulations using *L. pentosus* WSL5 and *T. ciferri* RSY53 as starter cultures, conducting *in vivo* assays to validate their enzyme inhibitory effects, and identifying and characterizing the genes responsible for these activities in the selected LAB and yeast strains.
